# Surface Treatment of Additively Manufactured Polyetheretherketone (PEEK) by Centrifugal Disc Finishing Process: Identification of the Key Parameters

**DOI:** 10.3390/polym16162348

**Published:** 2024-08-20

**Authors:** Jan Zentgraf, Florian Nützel, Nico Mühlbauer, Ulrich Schultheiss, Marius Grad, Thomas Schratzenstaller

**Affiliations:** 1 Laboratory for Medical Devices, Department of Mechanical Engineering, University of Applied Sciences Regensburg, 93053 Regensburg, Germanythomas.schratzenstaller@oth-regensburg.de (T.S.); 2 Regensburg Center of Health Sciences and Technology (RCHST), University of Applied Sciences Regensburg, 93053 Regensburg, Germany; 3 Regensburg Center of Biomedical Engineering (RCBE), University of Applied Sciences Regensburg, 93053 Regensburg, Germany; 4 Laboratory for Computer-Aided Engineering, Department of Mechanical Engineering, University of Applied Sciences Regensburg, 93053 Regensburg, Germany; florian.nuetzel@oth-regensburg.de; 5 Analytics Center, Department of Mechanical Engineering, University of Applied Sciences Regensburg, 93053 Regensburg, Germany; ulrich.schultheiss@oth-regensburg.de; 6 Laboratory for Material Science and Surface Analytics, Department of Mechanical Engineering, University of Applied Sciences Regensburg, 93053 Regensburg, Germany; marius.grad@oth-regensburg.de

**Keywords:** PEEK, fused deposition modeling, fused filament fabrication, centrifugal disc finishing, mass finishing, 3D printing, post-processing of 3D printed parts, surface treatment, high-performance polymers, additive manufacturing

## Abstract

Polyetheretherketone is a promising material for implants due to its good mechanical properties and excellent biocompatibility. Its accessibility to a wide range of applications is facilitated by the ability to process it with an easy-to-use manufacturing process such as fused filament fabrication. The elimination of disadvantages associated with the manufacturing process, such as a poor surface quality, is a main challenge to deal with. As part of the mass finishing process, centrifugal disc finishing has demonstrated good results in surface optimization, making it a promising candidate for the post-processing of additively manufactured parts. The objective of this study is to identify the key parameters of the centrifugal disc finishing process on the waviness of additively manufactured PEEK specimens, which has not been investigated previously. The waviness of the specimen was investigated by means of confocal laser scanning microscopy (CLSM), while weight loss was additionally tracked. Six parameters were investigated: type, amount and speed of media, use of compound, amount of water and time. Type of media, time and speed were found to significantly influence waviness reduction and weight loss. Surface electron microscopy images demonstrated the additional effects of deburring and corner rounding. Results on previous studies with specimens made of metal showed similar results. Further investigation is required to optimize waviness reduction and polish parts in a second post-processing step.

## 1. Introduction

High-performance polymers, such as polyetheretherketone (PEEK) or polyetherketoneketone (PEKK), are becoming increasingly prevalent in both industry and medical environments. Their chemical resistance and low density, coupled with their good mechanical properties and temperature resistance above 300 °C, set them apart from other polymers [1,2]. Additionally, their biocompatibility makes them suitable for use in a wide range of medical applications. A few examples for implants made of PEEK or PEKK include spinal cages, cranial and bone plates and dental implants [1,3,4,5,6,7]. The progressive ban on per- and polyfluoroalkyl substances (PFAS) in the EU and US and the subsequent search for suitable alternatives to fluoropolymers like polytetrafluoroethylen (PTFE), which is a highly prevalent material in the medical field, is a further contributing factor for a growing interest on high-performance polymers [8]. Given that PEEK is not a fluor polymer and exhibits promising properties, including high biocompatibility, excellent creep behavior, a modulus of elasticity comparable to that of bone and favorable tribological properties, it has the potential to serve as a substitute for PTFE in specific medical applications [1,9,10,11]. One of the many different methods to process high-performance polymers like PEEK is the fused filament fabrication (FFF) process. FFF is regarded as one of the most accessible and cost-effective 3D-printing technologies, although it does present certain limitations in terms of mechanical strength, surface quality and resolution [12,13]. Nevertheless, in the field of medicine, a number of implants have already been manufactured via the FFF process and successfully implanted [14,15]. All of these products take advantage of the mechanical properties of PEEK. However, for possible applications of PEEK in artificial joints, tribological properties are of importance. These properties are shown to be comparable to conventional highly cross-linked polyethylen [11]. The biocompatibility of PEEK and its proven suitability as an implant material are highly promising. Consequently, the question of whether a sliding surface made of PEEK is feasible for medical endoprosthesis is of significant relevance and has already been the subject of research in the case of conventionally manufactured PEEK surfaces [9,11,16,17]. However, there are a number of challenges due to the 3D-printing manufacturing process that need to be addressed. One limitation for components produced using the FFF method is the staircase effect, which is caused by the layer-by-layer deposition of polymer materials [18]. As the surface waviness strongly influences the tribological behavior, the staircase effect should be reduced for using FFF parts in sliding applications [19]. A variety of approaches exist for enhancing the surface quality of parts manufactured via the FFF process. Improvements to printing parameters or the implementation of chemical, mechanical or thermal post-processing methods represent a number of general techniques that have already been developed and tested. These include reducing layer height [20], vaporizing with acids [21], ironing [22] and milling [23], which have been identified as promising approaches for achieving high surface qualities. One of the many methods in the mechanical post-processing is the mass finishing process. This procedure is already well established in both the medical and automotive industries for its ability of deburring, raidiusing, removal of production residues and surface smoothing [24,25]. It also offers a high material removal rate that is ideal for removing surface irregularities caused by the manufacturing process [26]. As part of this mechanical finishing technique, centrifugal disc finishing offers several additional benefits, such as ease of use, speed of operation and low cost [27]. Due to the absence of any relevant studies including the centrifugal disc finishing process (CDFP) and PEEK parts produced by FFF, the objective is to examine the impact of centrifugal disc finishing on FFF-produced PEEK parts on waviness as an initial post-processing step and identify the most influential parameters to reduce the waviness caused by the manufacturing process.

## 2. Materials and Methods

### 2.1. 3D-Printing Specimen

**Figure 1:**
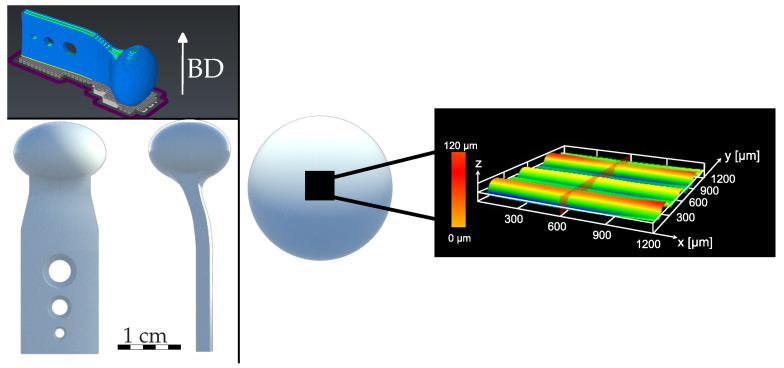
Illustration of specimen geometry and building direction (BD) on the left; the black rectangle shows the area of waviness measurement with unfiltered 3D height profile on the right.

The geometry used for the 3D printed sample has both concave and convex shapes with a round head on top (Figure 1). As the study is related to an additively manufactured temporomandibular joint (TMJ), this is the main reason for the shape of the specimen.

The selected geometry has the advantage of showing if the post-process can handle both shapes or if it is dependent on the geometry. Siemens NX CAD software (Version: 2015 (build: 2202), Siemens Industry Software Inc., Plano, TX, USA) was used for designing the specimen. Following the export of the geometry data as an STL file, they underwent transformation into 3D-printable data. Simplify3D (Simplify3D, LLC., Version: 5.1.2, Cincinnati, OH, USA) served as the slicing software, generating the necessary gcode file for printing. The prints were executed using the Apium M220 Fused Filament Fabrication (FFF) 3D printer (Apium Additive Technologies GmbH, M220, Karlsruhe, Germany). Apium PEEK 4000 Natural filament (diameter: 1.75 mm, density: 1.3 g/cm^3^, melt temperature: 340 °C ) was selected as the filament material. Consistency was maintained across all specimens by utilizing uniform parameters, including speed (2000 mm/min), nozzle temperature (485 °C), bed temperature (130 °C), number of perimeters (3) and infill (100%). Each print job produced four samples.

### 2.2. Centrifugal Disc Finishing Process

**Figure 2:**
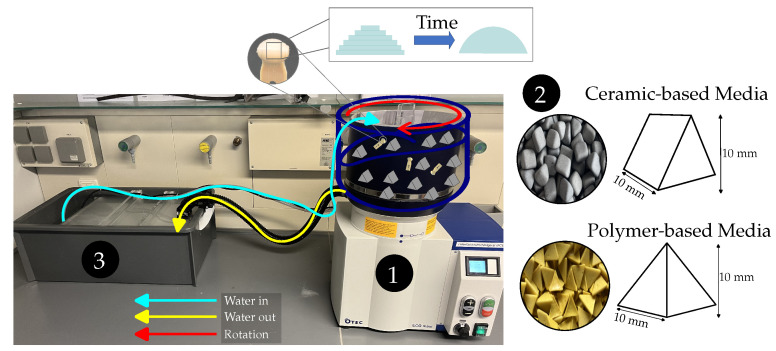
Illustration of CDFP. Reduction of staircase effect by time. Two different abrasive media (Nr.2) of different materials and different shapes. Ceramic media has prism shape, polymer media has pyramid shape. Water interaction between the machine (Nr.1) and the sedimentation box (Nr.3) shown with arrows.

The setup for carrying out the CDFP essentially consist of three components (Figure 2).

1. The centrifugal disc finishing machine (OTEC Präzisionsfinish GmbH, ECO-MAXI, Straubenhardt-Conweiler, Germany)
2. The abrasive media
3. A sedimentation box with a pump to reuse water

The centrifugal disc finishing machine comprises a bowl with stationary top and a rotating disc positioned at the base. The rotation of the disc initiates contact between the contents of the bowl, which includes the specimen, abrasive media and water-compound mixture, and drives this content in a helical path around the center of the bowl [28]. As is the case with all mass finishing operations, the impact and contact forces between the media and the specimen leads to material removal on the specimen surface [29]. Adjustable machine parameters are the amount of water flow, grinding time and speed. Two abrasive media were used in this study. The first was a prism-shaped ceramic-based media (Hoffmann Supply Chain GmbH, Garant 1010T Rough, Nuremberg, Germany) made of corundum. The second was a pyramid-shaped polymer-based media (Hoffmann Supply Chain GmbH, Garant 1010P Rough, Nuremberg, Germany) made of a polymer matrix with embedded corundum particles. The ceramic-based media are designed for high abrasiveness and high durability, while the polymer-based media are specifically intended for use on plastics. Both of these are claims made by the manufacturer. Given the different shape and type of material of the abrasive media, only the general influence of this parameter will be investigated. Furthermore, the amount of the media and the use of plastic compound (Hoffmann Supply Chain GmbH, Garant PLASTIC, Nuremberg, Germany) were selected as potentially influential parameters. Previous studies with metallic specimens showed additional parameters like slope angle of the disc, which were excluded as they could not be adjusted in the given setup [27]. Each grinding treatment was performed once, with three specimens post-processed at the same time. After each treatment, the machine, as well as the settling box, were cleaned and the abrasive media were replaced. Each treatment was performed with fresh abrasive media and fresh water-compound mixture.

### 2.3. Specimen Preparation

After both production and post-processing, a cleaning procedure was necessary to remove any residues of the respective processes. Therefore, an ultrasonic cleaner (Elma Schmidbauer GmbH, Elmasonic P60H, Singen, Germany) and an oven (Memmert GmbH + Co. KG, UF160 Plus, Schwabach, Germany), as well as cleaning agent and acetone, were used. The cleaning agent was prepared by mixing a ratio of 1:10 compound to water. Acetone was used to remove any polymer-media-based residue of the post-processing on the surface of the specimen, as the polymer media is soluble in acetone. For the cleaning process itself, constant frequency and curve types were used (frequency: 80 Hz, power: 100%, design: sweep). In order to ensure consistency, all specimens were cleaned the same way before each weight or surface measurement. The following procedure has proven itself in the course of several preliminary tests:

1. Ultrasonic cleaning for one hour at room temperature with acetone
2. Ultrasonic cleaning for four hours at 80 °C with cleaning agent
3. Ultrasonic cleaning for one hour with deionized water at 80 °C
4. Drying at 100 °C for eight hours

### 2.4. Weight Measurements

The weight of each specimen was measured before and after post-processing. The weight was determined by means of a precision scale (Sartorius, LA 310S, Göttingen, Germany), while each specimen was measured twice.

### 2.5. Surface Waviness Measurements

**Figure 3:**
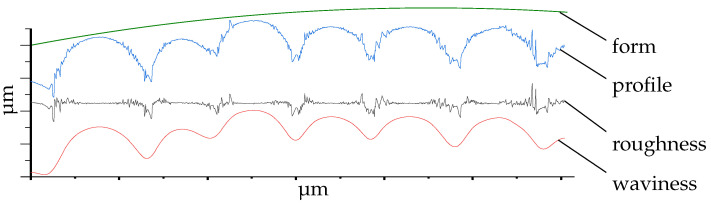
CLSM measurement filtered for different profiles; unfiltered primary profile (blue) represents actual measured data, including the waviness (red), roughness (black) and form (green) of the measured surface data.

Prior to post-processing and after cleaning, the surface waviness at the top of the specimen head (Figure 1) was examined. The measured area exhibited a variation of approximately 500 µm between the highest and lowest points, due to the rounding of the specimen head. Subsequently, for technical reasons, an optical method was employed in preference to a tactile one, such as atomic force microscopy. The selected method was a confocal laser scanning microscope (CLSM; Olympus, OLS4000, Tokyo, Japan), which has been demonstrated to be suitable in several previous studies [30,31,32,33,34]. For repeatability, a positioning guide was manufactured and used. This ensures the same area is measured (200 µm × 200 µm) before and after the treatment. Afterwards, the measured data were filtered for noises and inclination within the Olympus Lext software (Olympus, Lext OLS4000, Version: 2.2.6, Tokyo, Japan). In order to determine the waviness of the different samples, it is necessary to remove the λ_c_ and λ_f_ components of the primary profile of the measured surface [35]. These cutoff values were defined as λ_c_ = 80 µm and λ_f_ = 800 µm. The resulting waviness profile represents the long-wave component of the primary profile and was used to calculate the arithmetic mean, S_a_ (Figure 3).

### 2.6. Experimental Design

In order to examine the influence of different parameters of the CDFP on PEEK parts while using a minimum of samples and treatments, a statistical design method was selected. A definitive screening design (DSD) can show the mean and quadratic effects [36] and was designed by means of the software Minitab (Minitab 18, Minitab. Inc., State College, PA, USA). Six factors (type of media, speed, time, amount of water, use of compound and amount of media) were selected as input variables, while weight loss and waviness deviation were the response variables. The selection of these factors was based on previous findings [27] and the possibility of implementation within the given system. The number of treatments, n, was determined for an even number of factors, m, by n = 2m + 1 [37]. As three of the six factors (type of media, amount of water, use of compound) were determined as categorical, see Table 1, an additional center point and treatment was added. Consequently, the total number of treatments increased to 14. Even though the DSD is typically used with only continuous factors, it has been demonstrated that the inclusion of categorical variables is permissible [38,39]. Each treatment was conducted with three specimens, resulting in a total of 42 specimens being examined. Afterwards, the 3D-printed specimens were randomly distributed to the respective treatments.

To generate response variables for each treatment for weight loss as well as for waviness reduction, the mean value of each treatment (three specimens per treatment) was calculated. The deviation in percent from the initial value was then calculated according to formula (1).

(1) $$\mathrm{deviation}\;\left[\%\right]=\left(\frac{\mathrm{inital}\;\mathrm{value}-\mathrm{new}\;\mathrm{value}}{\mathrm{initial}\;\mathrm{value}}\right)*100$$

Using these response variables of waviness reduction and weight loss, an analysis of variance (ANOVA) was used to determine the statistical significance of each input factor. The normal distribution (Anderson–Darling test) of the residuals and the equality of variance (F-test or Bartlett’s test) were also checked as part of the ANOVA. As these two conditions are essential for the interpretation of the results, they had to be fulfilled [40]. If they were not met, the response variable was transformed using the Box–Cox transformation, which is an appropriate method for correcting non-normal distributions and non-equal variances [40,41].

## 3. Results and Discussion

### 3.1. Statistical Analysis

This study investigated the effect of six different CDFP parameters on the surface waviness reduction and weight loss of 3D-printed PEEK samples. Each response was statistically analyzed for its significant process parameters using an analysis of variance of the design of experiments scheme. The significance level was set at *p* = 0.05, so all *p*-values lower are considered a significant effect of the parameter.

Box–Cox transformation for both waviness reduction (λ=0.289022) and weight loss data (λ=0.0749978) were performed. Normally distributed data (waviness reduction: *p* = 0.076; weight loss: *p* = 0.242) and equality of variance were checked for all parameter–answer combinations. The ANOVA (Table 2) revealed significant main effects on waviness reduction for type of media (*p* = 0.000), speed (*p* = 0.014) and time (*p* = 0.037). For the purpose of analyzing weight loss, the same three main effects were found to be significant: type of media (*p* = 0.000), speed (*p* = 0.004) and time (*p* = 0.001). Both answer parameters show the same statistical significant parameters, while the process time has a higher influence on weight loss of the specimen.

**Figure A1:**
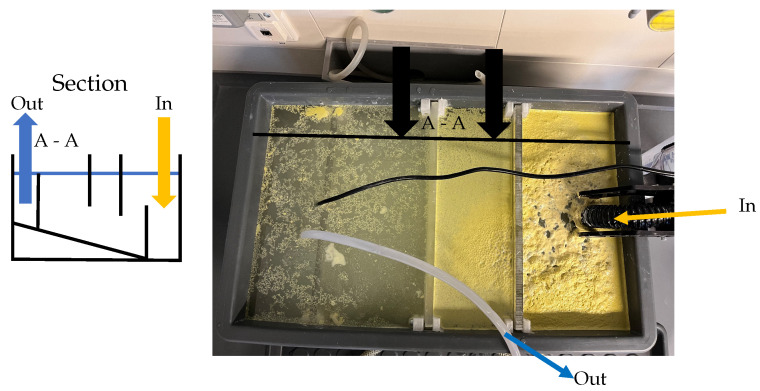
Schematic section view of sedimentation box. Foam formation as polymer media used without compound.

An investigation of the mass finishing process on aerospace parts made of a titanium alloy showed similar findings, with a high influence of the process time and media type. However, in this study the process time has an higher influence on the measured roughness value than the media type [42], which is vice versa to the results in Table 2. In their study, Djender and colleagues demonstrated that the speed and size of the abrasive media have a significant impact on the characteristics of metal surfaces. Additionally, they found that the amount of media has no notable influence on the outcome [27]. Our study on additively manufactured PEEK surfaces revealed the same results for waviness reduction. It may be that the results of metal surface treatment studies can be effectively transferred to polymer surface treatments. The use of compound and any quadratic effects have no significant influence on waviness reduction, nor on weight loss. However, even if ANOVA did not show a significant effect of compound on process outcome, the use of a compound is recommended. The use of plastic media in the absence of a compound resulted in the formation of excessive foam (Figure A1), which led to significant contamination of equipment and a high cleaning effort to prevent contamination in further treatments. Contamination with particles of previous treatments may compromise the stability of the process.

**Figure 4:**
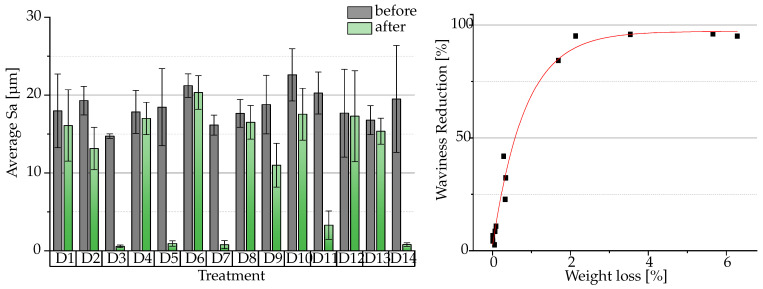
Absolute waviness of specimen (in S_a_) before and after centrifugal disc finishing (**left**). Exponential correlation of waviness reduction and weight loss (**right**).

An examination of Figure 4 reveals the significance of the type of media parameter. The mean S_a_ values differ drastically between the different treatments. In general, the waviness value of all specimens was reduced by post-processing, despite the differing amounts by which S_a_ changed.

**Figure 5:**
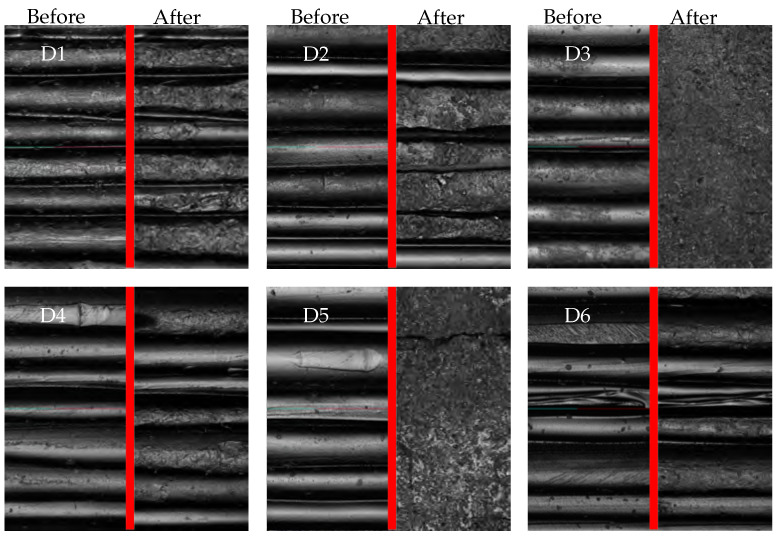

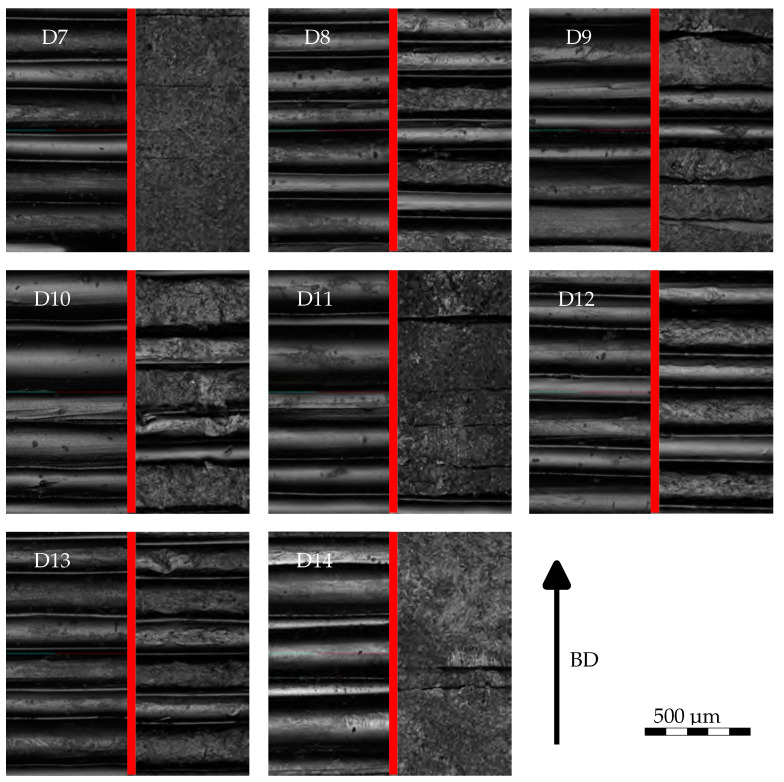
CLSM images of same area of each specimen before (**left**) and after (**right**) post-processing. The image was captured using a laser scanning microscope for specimens of Treatments D1 to D14. The results demonstrate varying waviness of surfaces.

The lowest S_a_ values relative to their initial values were obtained for D3, D5, D7, D11 and D14. The standard deviation for those treatment combinations was also reduced, indicating a leveling effect. These treatments were all performed with ceramic media, which suggests that the parameter type of media has an significant impact on the waviness reduction. Moreover, given that not all treatments utilizing ceramic-based media demonstrate comparable low values (D2 and D9), it appears that an additional factor may be responsible. Figure 5 visualizes the differences in surface quality depending on the process parameters per treatment and underpins the measured S_a_ values. Flattening the uneven surface of the sample caused by the printing process, the ceramic triangles are more abrasive than the polymer pyramids. Mean S_a_ values for all treatments with ceramic media are in the range of 0.58±0.17 µm (Treatment D2, Waviness Reduction: 32.26±9.34%) to 13.14±2.7 µm (Treatment D3: 96.70±0.10%) after post-processing. S_a_ values for treatment with polymer media are in a range of 15.35±1.68 µm (Treatment D12: 2.54±1.78%) to 20.33±2.17 µm (Treatment D10: 22.74±3.67%). The results of weight reduction and waviness reduction are reported for each result in Table A1.

As the choice of the medium (type, shape and size) is crucial for the surface characteristics, as evidenced by Holzknecht [24] and Kopp and Uhlmann [26], the significance of the type of media parameter in the present study is as expected. However, the individual influence of the shape and size of the media were not part of this investigation, being included in the type of media parameter.

In their study, Djender et al. found that a low or high rotational speed in a CFDP results in an increase in roughness. They recommended an optimum between minimum and maximum speed values [27]. As our study is observing the waviness reduction on a specimen with a distinctive staircase effect and not the roughness reduction, a high material removal is necessary. It was found that weight loss was exponentially correlated with low waviness values Figure 4. For treatments D9 and D11, an increase in speed (D9 = 200 rpm, D11 = 354 rpm) with equal significant parameters (type of media and time) led to an increased waviness reduction. This suggests that maximizing the speed parameter leads to a lower waviness.

### 3.2. Optical Analysis

Upon visual inspection of the images taken with a surface electron microscope (Zeiss, LEO 1455VP, Oberkochen, Germany), it becomes evident that the peaks are removed first during post-processing, followed by the valleys. The extent of material removal is contingent upon the abrasiveness of the medium and the treatment setup (process time, speed). When these parameters are sufficient, the surface exhibits a smooth and filled finish, lacking the typical waviness in FFF printing caused by layer-by-layer extrusion. Conversely, if the parameters are insufficient, the individual print layers remain visible. A further observation of the CDF process is the deburring and corner rounding effect of this post-processing technique. Sharp edges and burrs of the manufacturing process were rounded, whereof a small change in geometry is resulted. This has to be remembered in the case of high precision requirements. The lowest waviness was achieved with treatment D3, as illustrated in Figure 5, which compares the pre- and post-process states. The individual layers are no longer visible, and the resulting surface appears to be that of a bulk material.

**Figure 6:**
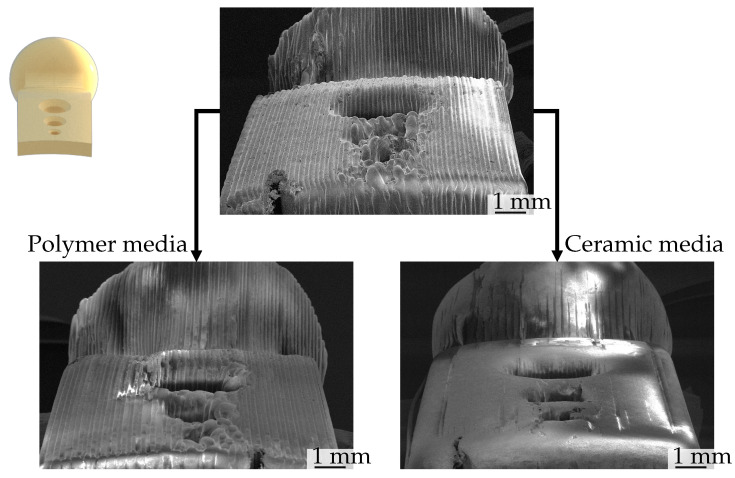
SEM images of specimen before and after grinding. Top middle shows the initial state after manufacturing and cleaning. Left is after post-processing with polymer-based media (Treatment D2); right shows after post-processing with ceramic media (Treatment D14).

A notable aspect of the optical analysis is the extent of post-processing, which is visible in Figure 6. While in some areas the various layers deposited during the manufacturing process are no longer visible, in others they remain unaltered. The unprocessed areas are located at the lower extremities and convex areas of the specimen. Longer processing, enhanced grinding performance or modification of the media geometry, which can also reach the narrow gaps, could result in a smoother and less wavy surface. It can be posited that a superior outcome of the manufacturing process may result in comparable post-processing outcomes across the entire specimen geometry.

The material removal on the surface of the specimens by centrifugal disc finishing has the potential to influence the surface properties and mechanical strength of the printed specimen. In the case of reinforced PEEK composites (for example, those containing carbon fibers), there is a possibility that the fibers may be exposed to excessive material removal. The formation of unfavourable fiber orientations in the material is a potential consequence. Moreover, for pure PEEK specimens, such as those employed in this study, there is a possibility of a disadvantageous impact on the interlayer bonding strength. This is due to the potential weakening by the material removal of the specimens, which could result in delamination of the individual layers. It is possible that this case may result in a complete failure of thin-walled printed parts, which were not included in the scope of this investigation.

## 4. Conclusions

Additively manufactured parts produced via the FFF process often exhibit significant surface waviness, which can compromise their functionality. To enhance the surface quality of these parts, post-processing techniques such as CDFP have proven to be effective. This study demonstrates that CDFP is a suitable method for improving the surface characteristics of 3D-printed PEEK parts, achieving a waviness reduction of up to 98%, even with non-optimized parameters. The study revealed that the type of media, speed and time parameters are significant factors influencing both surface waviness reduction and weight loss in 3D-printed PEEK specimens. Among these, the type of media parameter showed the most substantial impact on waviness reduction and weight loss. Correlated to the used abrasive media, it can be concluded that the ceramic-based media are more effective than the polymer-based ones. In comparison to prior studies [27,42] on metal surfaces, these results of significant parameters are nearly consistent. However, contrary to the metal treatment studies, this research found that media type had a more significant effect than process time on waviness reduction. Although the study found no significant effect of the use of compound on waviness reduction or weight loss, it recommends the use of compound for process stability reasons. Another finding was the exponential correlation between weight loss and waviness reduction, indicating that a more aggressive material removal leads to less wavy surfaces. Furthermore, the study demonstrated that a smoother surface after the manufacturing process will result in a more even surface after post-processing, emphasizing the necessity for optimal manufacturing parameters. Future studies should investigate the effects of the CDFP technique on PEEK composites like carbon-fiber-reinforced PEEK and on thinner-walled or less dense printed parts, as the current study focused on fully dense pure PEEK specimens. The study highlights the importance of selecting the appropriate media type and optimizing process parameters to achieve the desired surface characteristics.

In conclusion, the research demonstrates that careful control of the three parameters of type of media, speed and time in the centrifugal disc finishing process can significantly enhance the surface quality of 3D-printed PEEK parts. This has practical implications for improving the performance of the finished product.

## Figures and Tables

**Table 1:** Design of experiment as definitive screening design with six factors.

Treatment	Type of Media ^1^	Speed [rpm]	Time [min]	Amount of Water ^2^	Use of Compound	Amount of Media [dm^3^]
D1	P	354	240	max	Yes	1.50
D2	C	200	30	min	No	1.00
D3	C	277	240	min	No	1.50
D4	P	277	30	max	Yes	1.00
D5	C	354	135	max	No	1.00
D6	P	200	135	min	Yes	1.50
D7	C	200	240	min	Yes	1.00
D8	P	354	30	max	No	1.50
D9	C	200	30	max	No	1.50
D10	P	354	240	min	Yes	1.00
D11	C	354	30	min	Yes	1.25
D12	P	200	240	max	No	1.25
D13	P	277	135	min	No	1.25
D14	C	277	135	max	Yes	1.25

^1^ P = Polymer; C = Ceramic. ^2^ min = 24 mL/s; max = 32 mL/s.

**Table 2:** Analysis of variance (ANOVA) after response transformation (Box–Cox) for the responses of waviness reduction and weight loss.

Source	DF	Seq SS	Contribution	Adj SS	Adj MS	F-Value	*p*-Value
Waviness reduction	9	0.822052	98.32%	0.822052	0.091339	26.08	0.003
Linear	6	0.815685	97.56%	0.815685	0.135948	38.82	0.002
Type of media ^★^	1	0.696120	83.26%	0.732762	0.732762	209.26	0.000
Speed ^★^	1	0.063718	7.62%	0.060971	0.060971	17.41	0.014
Time ^★^	1	0.044375	5.31%	0.033081	0.033081	9.45	0.037
Amount of Water	1	0.004870	0.58%	0.002864	0.002864	0.82	0.417
Use of compound	1	0.006485	0.78%	0.005998	0.005998	1.71	0.261
Amount of media	1	0.000118	0.01%	0.000118	0.000118	0.03	0.864
Square	3	0.006367	0.76%	0.006367	0.002122	0.61	0.645
Speed × Speed	1	0.003988	0.48%	0.002623	0.002623	0.75	0.436
Time × Time	1	0.001868	0.22%	0.002234	0.002234	0.64	0.469
Amount of media × Amount of media	1	0.000512	0.06%	0.000512	0.000512	0.15	0.722
Error	4	0.014007	1.68%	0.014007	0.003502		
Total	13	0.836059	100.00%				
Weight loss	9	0.332640	99.21%	0.332640	0.036960	55.70	0.001
Linear	6	0.324430	96.76%	0.324430	0.054072	81.49	0.000
Type of media ^★^	1	0.238061	71.00%	0.261453	0.261453	394.02	0.000
Speed ^★^	1	0.022923	6.84%	0.025191	0.025191	37.96	0.004
Time ^★^	1	0.060929	18.17%	0.056329	0.056329	84.89	0.001
Amount of water	1	0.000636	0.19%	0.000441	0.000441	0.66	0.461
Use of compound	1	0.000087	0.03%	0.000272	0.000272	0.41	0.557
Amount of media	1	0.001795	0.54%	0.001795	0.001795	2.70	0.175
Square	3	0.008210	2.45%	0.008210	0.002737	4.12	0.102
Speed × Speed	1	0.002786	0.83%	0.000502	0.000502	0.76	0.434
Time × Time	1	0.004508	1.34%	0.003404	0.003404	5.13	0.086
Amount of media × Amount of media	1	0.000916	0.27%	0.000916	0.000916	1.38	0.305
Error	4	0.002654	0.79%	0.002654	0.000664		
Total	13	0.335294	100.00%				

^★^ Significant Parameter.

## Data Availability

All data are contained within the article.

## Abbreviations

The following abbreviations are used in this manuscript:

DSD	Definitive Screening Design
DOE	Design of Experiment
PEEK	Polyetheretherketone
PEKK	Polyetherketoneketone
CDFP	Centrifugal Disc Finishing Process
FFF	Fused Filament Fabrication
SEM	Scanning Electron Microscope
CLSM	Confocal Laser Scanning Microscope
ANOVA	Analysis of Variance
TMJ	Tempormandibular Joint

## Appendix B

**Table A1:** Weight reduction and waviness reduction of each treatment.

Treatment	Weight Loss [%]	Waviness Reduction [%]
D1	0.09	10.82
D2	0.34	32.27
D3	5.66	96.06
D4	0.01	4.32
D5	6.28	95.04
D6	0.02	4.25
D7	2.14	95.11
D8	0.01	6.65
D9	0.29	41.81
D10	0.33	22.75
D11	1.69	84.30
D12	0.05	2.54
D13	0.07	8.53
D14	3.54	95.86
